# Tree diversity and functional leaf traits drive herbivore‐associated microbiomes in subtropical China

**DOI:** 10.1002/ece3.7434

**Published:** 2021-03-31

**Authors:** Yi Li, Douglas Chesters, Ming‐Qiang Wang, Tesfaye Wubet, Andreas Schuldt, Perttu Anttonen, Peng‐Fei Guo, Jing‐Ting Chen, Qing‐Song Zhou, Nai‐Li Zhang, Ke‐Ping Ma, Helge Bruelheide, Chun‐Sheng Wu, Chao‐Dong Zhu

**Affiliations:** ^1^ Key Laboratory of Zoological Systematics and Evolution Institute of Zoology Chinese Academy of Sciences Beijing China; ^2^ College of Biological Sciences University of Chinese Academy of Sciences Beijing China; ^3^ Department of Community Ecology Helmholtz Centre for Environmental Research Halle/Saale Germany; ^4^ Forest Nature Conservation Georg‐August‐University Göttingen Göttingen Germany; ^5^ Institute of Biology/Geobotany and Botanical Garden Martin Luther University Halle‐Wittenberg Halle Germany; ^6^ German Centre for Integrative Biodiversity Research (iDiv) Halle‐Jena‐Leipzig Leipzig Germany; ^7^ College of Plant Protection Yunnan Agriculture University Yunnan China; ^8^ Research Center of Forest Management Engineering of State Forestry and Grassland Administration Beijing Forestry University Beijing China; ^9^ State Key Laboratory of Integrated Pest Management Institute of Zoology Chinese Academy of Sciences Beijing China; ^10^ State Key Laboratory of Vegetation and Environmental Change Institute of Botany Chinese Academy of Sciences Beijing China

**Keywords:** 16S rRNA, Bacteria, BEF‐China, herbivore‐associated microbiome, leaf characteristics, Lepidoptera, Microbial ecology

## Abstract

Herbivorous insects acquire microorganisms from host plants or soil, but it remains unclear how the diversity and functional composition of host plants contribute to structuring herbivore microbiomes. Within a controlled tree diversity setting, we used DNA metabarcoding of 16S rRNA to assess the contribution of Lepidoptera species and their local environment (particularly, tree diversity, host tree species, and leaf traits) to the composition of associated bacterial communities. In total, we obtained 7,909 bacterial OTUs from 634 caterpillar individuals comprising 146 species. Tree diversity was found to drive the diversity of caterpillar‐associated bacteria both directly and indirectly via effects on caterpillar communities, and tree diversity was a stronger predictor of bacterial diversity than diversity of caterpillars. Leaf toughness and dry matter content were important traits of the host plant determining bacterial species composition, while leaf calcium and potassium concentration influenced bacterial richness. Our study reveals previously unknown linkages between trees and their characteristics, herbivore insects, and their associated microbes, which contributes to developing a more nuanced understanding of functional dependencies between herbivores and their environment, and has implications for the consequences of plant diversity loss for trophic interactions.

## Graphical abstract

In this study, we hypothesized that the community composition of herbivore‐associated microbes is driven by specific aspects of their surroundings (particularly, leaf traits, host tree species, and diversity thereof) and by the composition and diversity of the host herbivore species. We found that tree diversity and leaf traits drive caterpillar‐associated microbes in a controlled setting. It revealed the linkages between tree (leaves), herbivores, and associated microbes, which contributes to the development of a more comprehensive understanding of relationships and functional dependencies between herbivores and their environment, and of the consequences of plant diversity loss for trophic interactions.

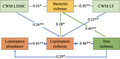

## 1 INTRODUCTION

Insect symbionts comprise of bacteria, fungi, and viruses, and persist both in the insects and on the cuticle of their exoskeleton and engage in a variety of interactions (Frago et al., 2012; Klepzig & Six, 2004). Herbivorous insects can acquire specific symbiont bacterial species from host plants or environment, such as the soil in which the host plants grow (Kikuchi et al., 2012; Sugio et al., 2015). Many symbionts found in the gut of insects are allied to microorganisms of the immediate environment (Frago et al., 2012). In addition to gut symbionts, herbivorous insects are known to acquire intra‐ and extracellular microbes also from host plants (Caspi et al., 2011; Li et al., 2016).

Studies of bacterial communities of Lepidoptera have increased rapidly in past several years, mostly because Lepidoptera possess extraordinary species richness and are important forest and agricultural pests (Belda et al., 2011; Broderick et al., 2004; Gayatri et al., 2012; Xia et al., 2013; Xiang et al., 2006). However, some recent studies reported that caterpillars lack resident gut microbes (Hammer et al., 2017; Hammer et al., 2019). Most of the microbes found in the Lepidoptera gut were found to be shared with the surrounding leaf surface or with the soil in which the host plant grew, and the bacteria seem to have no effects on growth and survival of the caterpillar (Hammer et al., 2017; Whitaker et al., 2016). If indeed lacking a persistent microbiome, then any microbial‐driven processes in caterpillars might be more susceptible to environmental influences than would otherwise be.

**FIGURE 1:**
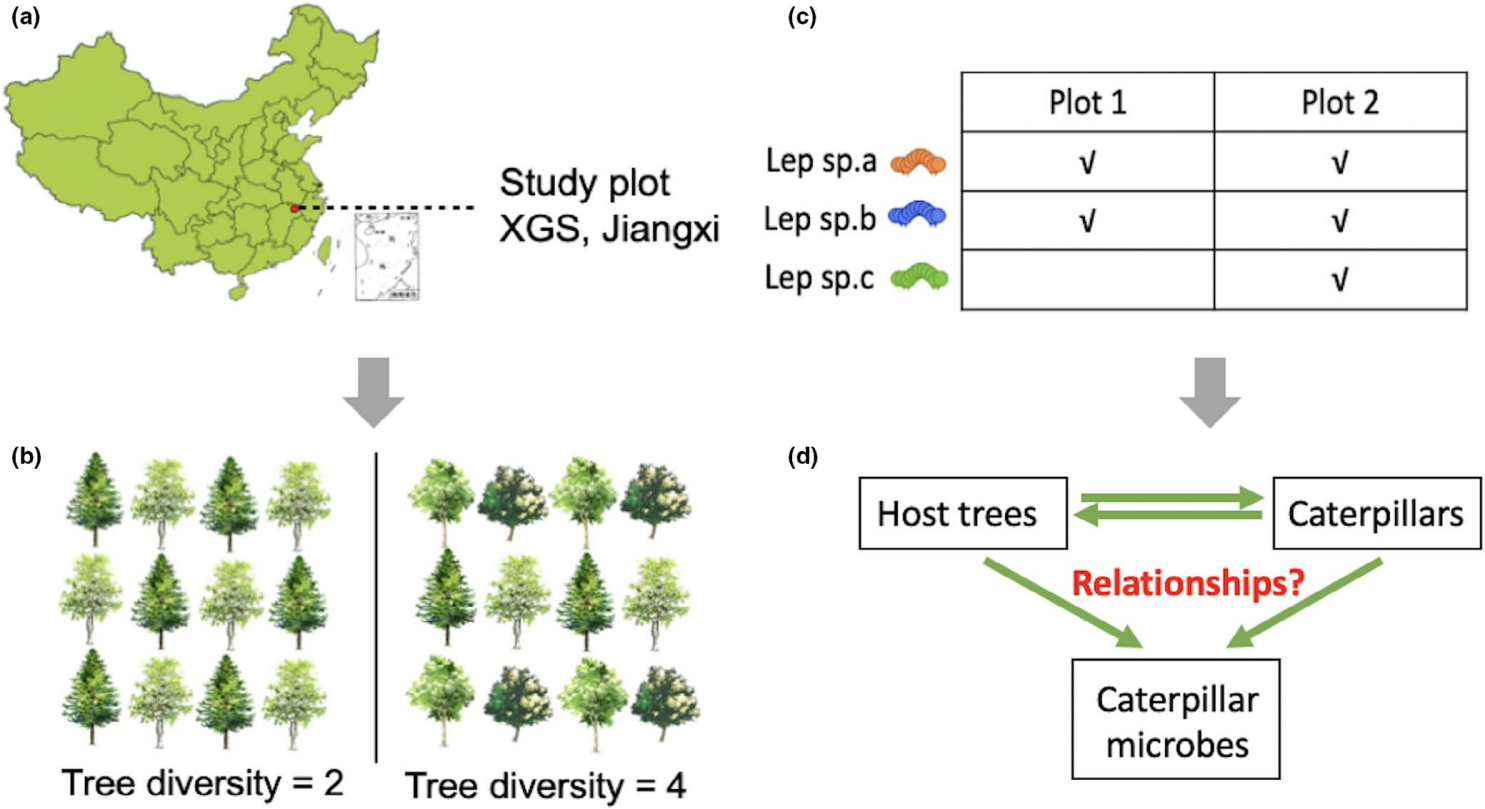
Overview of the study; (a) location of the study site; Xin‐Gang mountain, Jiangxi Province (29°08′–29°11′N, 117°90′–117°93′E), southeast China, with a typical subtropical climate; (b) two example plots in the study site, with tree species richness of 2 & 4; (c) presence / absence of three Lepidoptera species in the two plots; (d) the relationships between trees, lepidopteran samples and their associated bacterial OTUs

What kind of environmental factors influence the microbial community of herbivorous insects? And to what extent did these factors influence the herbivore microbes? To answer these questions, we analyze the relationships between the bacterial composition and diversity of host plant and herbivores. This is conducted in the “BEF‐China” experiment, a large‐scale forest biodiversity experiment incorporating random extinction scenarios of tree species, and used to estimate the ecological effects of biodiversity loss (Bruelheide et al., 2014). We target microbial symbionts on and in the caterpillar body, using 16S rRNA gene sequencing (Figure 1). We hypothesize that the community composition of herbivore‐associated microbes is driven by specific aspects of their surroundings (i.e., leaf traits, host tree species identity, and diversity of tree species in the host tree community) and by the composition and diversity of the host herbivore species. We aim to assess the relative importance of direct host‐mediated effects versus environmentally mediated effects on the herbivore microbiome.

## 2 MATERIALS AND METHODS

### 2.1 Experimental design for study sites

The study was conducted in the BEF‐China forest biodiversity experiment, which was established in the southeast of China (Xingangshan, Jiangxi Province, 29°08′–29°11′N, 117°90′–117°93′E) in 2009. The study area has a subtropical monsoon climate with an annual mean temperature of 16.7°C and precipitation of 1,821 mm (Yang et al., 2013). The 38.4 ha study area consists of two sites including a total of 566 plots (25.8 *25.8 m/plot, 271 plots in site “A” and 295 plots in site “B”). In each plot, 400 trees were planted in 20 rows and 20 columns. The species pool includes 40 species of trees. Species were selected for each plot according to a random broken stick design for extinction scenarios of 24, 16, 8, 4, and 2 mixtures and monocultures (the 24‐species mixtures are an additional treatment on top of this design; Bruelheide et al., 2014).

### 2.2 Sample collection of individual caterpillars

We focus on the larval stage, being the primary feeding stage of Lepidoptera and typically the focus for studies of herbivory. The collection of lepidopteran larvae used herein has been previously reported, with focus on tree diversity effects on herbivores themselves in Wang et al. (2019, 2020); thus, here we focus on the microbiomes associated with these herbivores. In October 2018, we selected individuals from the caterpillar samples for extraction of bacterial DNA. We recognize that DNA contamination could be an issue. We therefore took steps to avoid this. Firstly, the caterpillar samples were cleaned with sterile water and 75% ethanol and then kept in separate tubes with 99.5% ethanol. Secondly, we extracted whole lepidopteran larvae, but we broke their bodies sufficiently that we can obtain their gut microbiota. All individuals were stored in a − 20 ℃ freezer prior to DNA extraction. To ensure the comparability of caterpillar‐associated bacteria across the tree diversity levels, we selected the individual caterpillars randomly from different plots based on the BEF‐China design (118 caterpillars came from monocultures, and 110, 104, 97, 98, and 107 came from the mixtures of 2, 4, 8, 16, and 24 species, respectively; details see from Table 1). Those Lepidoptera species that had the highest abundances in each plot were selected as representative for the plot. As the number of caterpillars among plots and that of different Lepidoptera species varied greatly, we selected the caterpillars to sample as many different tree species per plot as possible. Because of tree mortality, the whole sample included 37 tree species from 54 plots (Table 1). For each plot, all bacterial sequences obtained from the selected caterpillars served as the bacterial community of the Lepidoptera larvae from the given plot. Totally, this resulted in the selection of 444 trees covering the full tree diversity gradient and a total of 634 caterpillar individuals.

**TABLE 1:** Tree species richness, composition, and caterpillar sample size of the study plots

Site	Plot	Tree richness	Caterpillar number	Tree Species Composition
A	E31	1	5	*Quercus fabri*
A	E33	1	5	*Lithocarpus glaber*
A	E34	1	5	*Castanea henryi*
A	F21	1	5	*Quercus serrata*
A	G24	1	5	*Koelreuteria bipinnata*
A	I28	1	5	*Liquidambar formosana*
A	K19	1	4	*Schima superba*
A	L11	1	5	*Castanopsis sclerophylla*
A	N11	1	5	*Sapindus mukorossi*
A	N13	1	4	*Sapium sebiferum*
A	O22	1	4	*Cyclobalanopsis myrsinifolia*
A	O27	1	5	*Choerospondias axillaris*
A	R14	1	5	*Cyclobalanopsis glauca*
A	W14	1	5	*Nyssa sinensis*
A	C32	2	9	*C. henryi; N. sinensis*
A	H31	2	10	*L. formosana; S. mukorossi*
A	I27	2	10	*C. axillaris; S. sebiferum*
A	J21	2	10	*K. bipinnata; L. glaber*
A	P23	2	4	*S. superba; R. chinensis*
A	P26	2	7	*Q. serrata; C. sclerophylla*
A	Q21	2	6	*Q. fabri; C. glauca*
A	F27	4	18	*Q. serrata; Ch. axillaris; S. sebiferum; C. sclerophylla*
A	N8	4	19	*S. superba; Q. fabri; Rh. chinensis; C. glauca*
A	P19	4	12	*C. henryi; L. formosana; S. mukorossi; N. sinensis*
A	W/X12	4	10	*K. bipinnata; C. myrsinifolia; L. glaber; C. eyrei*
A	S10	8	29	*C. henryi; L. formosana; Q. serrata; C. axillaris; S. mukorossi; N. sinensis; S. sebiferum; C. sclerophylla*
A	T15	8	34	*S. superba; K. bipinnata; Q. fabri; R. chinensis; C. glauca; C. myrsinifolia; L. glaber; C. eyrei*
A	L22	16	60	*C. henryi; S. superba; L. formosana; Q. serrata; K. bipinnata; Q. fabri; C. axillaris; R. chinensis; C. glauca; S. mukorossi; N. sinensis; S. sebiferum; C. myrsinifolia; L. glaber; C. eyrei; C. sclerophylla*
A	N9	24	54	All 16 species + additional 8 species for Site A^a^
B	I25	1	4	*Manglietia yuyuanensis*
B	M7	1	5	*Betula luminifera*
B	N28	1	5	*Idesia polycarpa*
B	N5	1	5	*Ailanthus altissima*
B	Q27	1	4	*Alniphyllum fortunei*
B	Q29	1	5	*Machilus leptophylla*
B	R29	1	5	*Castanopsis fargesii*
B	U16	1	5	*Elaeocarpus japonicus*
B	V24	1	5	*Elaeocarpus chinensis*
B	W10	1	5	*Phoebe bournei*
B	W11	1	4	*Elaeocarpus glabripetalus*
B	G28	2	10	*B. luminifera; C. fargesii*
B	M24	2	8	*C. biondii; E. glabripetalus*
B	M29	2	10	*E. japonicus; P. bournei*
B	O27	2	9	*M. yuyuanensis; Q. phillyreoides*
B	V19	2	9	*E. chinensis; M. thunbergii*
B	V23	2	8	*A. fortunei; M. leptophylla*
B	M22	4	8	*A. fortunei; E. chinensis; M. thunbergii; M. leptophylla*
B	O31	4	14	*C. biondii; E. glabripetalus; E. japonicus; P. bournei*
B	R3	4	14	*B. luminifera; C. fargesii; M. yuyuanensis; Q. phillyreoides*
B	S18	4	7	*A. altissima; I. polycarpa; M. flexuosa; M. grijsii*
B	J29	8	21	*A. altissima; C. biondii; E. glabripetalus; E. japonicus; I. polycarpa; M. flexuosa; M. grijsii; P. bournei*
B	Q17	8	13	*A. fortunei; B. luminifera; C. fargesii; E. chinensis; M. thunbergii; M. leptophylla; M. yuyuanensis; Q. phillyreoides*
B	S22	16	38	*A. altissima; A. fortunei; B. luminifera; C. fargesii; C. biondii; E. chinensis; E. glabripetalus; E. japonicus; I. polycarpa; M. flexuosa; M. grijsii; M. thunbergii; M. leptophylla; M. yuyuanensis; P. bournei; Q. phillyreoides*
B	T8	24	54	All 16 species + additional 8 species for Site B^b^

^a^ Additional species for Site A: *Sapium discolor, Castanopsis carlesii, Diospyros glaucifolia, Melia azedarach, Acer davidii, Daphniphyllum oldhamii, Quercus acutissima, Cinnamomum camphora*

^b^ Additional species for Site B: *C. eyrei, C. sclerophylla, C. camphora, C. glauca, D. oldhamii, D. glaucifolia, L. glaber, S. superb*

As the current strategy for sampling caterpillar‐associated bacteria at the plot level has some limitations (particularly, the bacterial composition of some rare Lepidoptera species was not considered), we tested whether the results were affected by sample size. To this end, we used additional linear models to check the relationships between bacterial and tree species richness at the tree richness level (Figure 3a and 3b). We found that the different Lepidoptera species feeding on the same tree species showed similar bacterial communities (relative abundance of bacterial phyla; Figure S1), and there was no significant difference in bacterial diversity (results not shown).

### 2.3 DNA extraction, amplification, quantitation, and sequencing

Total DNA was extracted from the individual caterpillars (because many larvae were very small or less than 5 mm) using Qiagen DNeasy Tissue Kit (QIAGEN GmbH, Hilden, Germany), following the manufacturer's protocol. Samples were processed using sterile tools and conditions. The DNA extracts were quantified using the Qubit 4.0 Fluorometer and stored at −20°C for further processing.

The V3 and V4 regions of the 16S rRNA gene, a fragment 468 bp in length, were targeted as it has among the highest taxonomic coverage in bacteria (Klindworth et al., 2012). V3 and V4 were amplified using the 16S forward (5′ ‐ACTCC TACGG GAGGC AGCAG −3′) and reverse (5′ ‐ GGACT ACNVG GGTWT CTAAT ‐ 3′; Zeng et al., 2011) primers. The reaction system involved 4 μL of 5 × FastPfu Buffer, 2 μL of 2.5 mM dNTPs, 0.8 μL of forward primer, 0.8 μL of reverse primer, 0.4 μL of FastPfu Polymerase, 0.2 μL of BSA, 9.5 μL of water, and 10 ng of template DNA. The conditions of the PCR were 3 min template denaturation at 95°C, followed by 30 cycles at 95°C for 30 s per cycle, 30 s annealing at 53°C, elongation at 72°C for 45 s, and 10 min extension at 72°C finally. The resulting PCR products were extracted from 2% agarose gel and further purified using the AxyPrep DNA Gel Extraction Kit (Axygen Biosciences, Union City, CA, USA), and quantified using QuantiFluor™‐ST (Promega, USA) according to the manufacturer's protocol.

Caterpillar‐derived amplicons were purified and barcoded (Wang et al., 2019, 2020). Purified amplicons were quantified, pooled in equimolar, and paired‐end sequenced using the V2 Illumina chemistry (2x300 bp) on an Illumina MiSeq platform (Illumina, San Diego, USA; Illumina, Inc. 2015) according to the standard protocols by Majorbio Bio‐Pharm Technology Co. Ltd. (Shanghai, China).

### 2.4 Bioinformatics analyses

We used both VSEARCH v 2.8.1 (Rognes et al., 2016) and USEARCH v 11 (Edgar, 2010) to process raw sequences. Firstly, the read pairs were merged, primers were trimmed, and quality filtering excluded short and low‐quality reads (below 25), using VSEARCH. Then, we dereplicated the data (retaining abundance information) and generated OTUs through the UNOISE algorithm (≥ 100%) in USEARCH (Edgar, 2016). Bacterial abundances are calculated after denoising by generating an OTU table with otutab command. Finally, the OTUs were assigned taxonomic information using the Silva 138 SSU database by using the “sintax” command in USEARCH (threshold ≥ 90%), after which nonbacteria OTUs were removed (Pruesse et al., 2007). Bacteria were classified at the level of phylum, class, order, family, genus, and species.

Phylogenetic diversity (PD) of bacteria was incorporated as response variables. To construct the bacterial phylogeny, we used MAFFT v 7.0 (Misawa et al., 2002) to align the sequences, trimmed the alignment with MEGA v 7.0 (Kumar et al., 2016), and inferred the phylogeny using the ML software IQ‐TREE v 1 (Nguyen et al., 2015).

### 2.5 Leaf traits

We selected 11 morphological and chemical leaf traits which we considered prime candidates for determining leaf quality for herbivore insects, and to characterize plot conditions in accordance with nutritional quality and potential defense traits of the trees. We used leaf area, specific area, dry matter content and toughness as the main morphological traits, and leaf potassium, calcium, magnesium, sodium, phosphorus, carbon, and nitrogen content, and C: N ratio, as the chemical leaf traits (see Table S1 for abbreviations that will be used below). All of these traits were measured on sun‐exposed, fully expanded, undamaged leaves from five to seven individuals per tree species according to standard protocols (Kröber et al., 2012; Pérez‐Harguindeguy et al., 2003). More details on trait measurements can be found in Kröber et al. (2012).

### 2.6 Community‐weighted mean trait values, functional and phylogenetic diversity

We used the community‐weighted mean (CWM) of each trait as well as the functional diversity of selected traits for each tree species, which is the mean value of each species’ trait weighted by the species contribution to the plot wood volume. The CWM values of each trait in each plot were calculated by the following equation: $\mathrm{CW}M_{\mathrm{tp}}=\sum_{i=1}^{S}V_{\mathrm{ip}}\times t_{i}$ where $V_{\mathrm{ip}}$ is the relative tree wood volume of species *i* in plot p and $t_{i}$ is the mean trait value of species *i* (Garnier et al., 2004). Tree wood volume was estimated from basal area and tree height measured on trees in the center of each plot according to Fichtner et al. (2017). We used species‐mean trait values as previous studies in BEF‐China demonstrated that variability in trait–environment relationships was much more pronounced at the interspecific than the intraspecific level (Schuldt et al., 2012).

To characterize the plot conditions of the study sites, the following metrics were calculated at the plot level. The functional diversity of trees was calculated by the mean pairwise distance of trait values among tree species, weighted by relative wood volume, and expressed as Rao's Q (Ricotta & Moretti, 2011). We also depicted PD of tree communities by wood volume‐weighted phylogenetic mean pairwise distance (MPD), which in the abundance‐weighted case is equivalent to Rao's Q (Tucker et al., 2016). In addition, we calculated the mean nearest taxon distance (MNTD), a measure of the phylogenetic distance to the nearest taxon, which for each taxon quantifies the extent of terminal clustering (Webb, 2000; Webb et al., 2002). Phylogenetic indices were calculated on a maximum likelihood phylogenetic tree of all woody species recorded in all plots (Purschke et al., 2017).

In addition to plants, indices of Lepidoptera diversity were included. We incorporated Faith's PD, abundance‐weighted phylogenetic MPD and MNTD of the lepidopteran communities sampled per study plot (Wang et al., 2019) as predictors in our models. The phylogenetic data were obtained from a maximum likelihood phylogenetic tree based on all lepidopteran samples we collected from 2017 and 2018 (Wang et al., 2020).

### 2.7 Statistical analyses

Statistical analyses were conducted using the packages picante (Kembel et al., 2010), vegan (Oksanen, 2008), ape (Paradis & Schliep, 2019), edgeR (Robinson et al., 2010), phyloseq (McMurdie & Holmes, 2013), lavaan (Rosseel, 2012), and lulu (Frøslev et al., 2017) in R v 3.5.2 (http://www.R‐project.org). Firstly, to eliminate the impacts on differing read numbers across samples, the number of sequences of all samples was rarefied to the lowest read number by using the “rarefy” function of the vegan package (rarify depth: 11,000). The bacterial S_obs_ (observed bacterial richness), Chao1 (nonparametric estimator for bacterial richness), Shannon diversity, and Pielou's evenness were calculated for each plot from bacterial abundance using the “diversity” function of the vegan package. To improve normality and variance in homogeneity of the model residuals, tree species richness, Lepidoptera richness, abundance, and bacterial S_obs_ were log‐transformed, and Chao1, Shannon diversity, and Pielou's evenness were square‐root transformed. All continuous predictors were standardized before the analyses.

To avoid multicollinearity affecting our statistical analyses, we tested correlations among all predictors through the Pearson's correlation coefficients (r > 0.7 interpreted as a strong correlation) and examined variance inflation factors (VIF) in statistical models. Single regression analyses were first used to assess the relationships between species richness, herbivory, phylogenetic metrics of the diversity of trees/lepidopteran larvae (PD, MPD, and MNTD) and alpha diversity of the bacterial community. Then, we used linear models to test the potential effects of tree species richness, Lepidoptera richness, leaf traits, and plot covariables on caterpillar‐associated bacteria. We used bacterial richness (S_obs_ and Chao1), bacterial PD, Shannon diversity, and Pielou's evenness as response variables. For predictors, we included tree species richness, MPD, MNTD, tree functional diversity, CWMs of the selected leaf traits and woody volume, Lepidoptera richness, abundance, MPD, and MNTD. We did not include Lepidoptera richness and phylogenetic diversity in the same models because of their strong collinearity (Pearson's r > 0.9, *p* <.001). The same applied to tree species richness and MPD (Pearson's r > 0.7, *p* <.001). In addition, we used the interaction between site and tree species richness/tree functional diversity in our models. The linear models were simplified in a stepwise procedure until we obtained the model with the lowest AICc.

Path analyses were conducted to explore the potential causal relationships among tree species richness, Lepidoptera richness, leaf traits, plot covariables, and richness of caterpillar‐associated bacteria. Based on prior and theoretical knowledge, we hypothesized that species richness of trees and Lepidoptera might directly influence bacterial communities. In addition, tree species richness could also indirectly influence bacterial communities via Lepidoptera richness. Concurrently, leaf traits may have direct or indirect effects on bacterial communities. Nonsignificant pathways were gradually removed if their removal improved model fit (Scherber et al., 2010). The model fit was assessed by comparative p value, fit index value (CFI), Akaike Information Criteria (AIC), and root mean square errors of approximation (RMSEA). Adequate model fits are indicated by high CFI, low AIC, and low RMSEA.

As for bacterial Beta diversity, we defined it as Arrhenius exponent “z” (calculated with “betadiver” function) in our study and compared the variances of dissimilarities using the “adonis” function in vegan (Anderson, 2006), with a p value obtained from 9,999 permutations. Moreover, we used a Mantel test to check whether bacterial composition was influenced by spatial location. Differentially abundant bacterial OTUs were detected using edgeR’s generalized linear model (GLM) approach. This method allows for testing differential bacterial abundance between different levels of factors by employing a design matrix to account for complex experimental designs. We fitted a generalized linear model with a negative binomial distribution to the normalized values for each of the bacterial OTUs. Differential abundance was tested using a likelihood ratio test, using an adjusted P value cutoff of 0.01. The bacterial OTUs that were enriched in mixtures were compared with bacterial counts from the monoculture plots as reference, and in addition, for higher species richness, also with the bacterial counts from 2, 4, and 8 species mixtures. Distance‐based redundancy analysis (db‐RDA analysis) based on Bray–Curtis distances was performed using the function “capscale” from the R Package vegan. To determine whether plot covariables and CWM of leaf traits contribute to explaining the microbial community structure of lepidopteran samples, we applied variance partitioning, based on the db‐RDA analysis.

## 3 RESULTS

**FIGURE 2:**
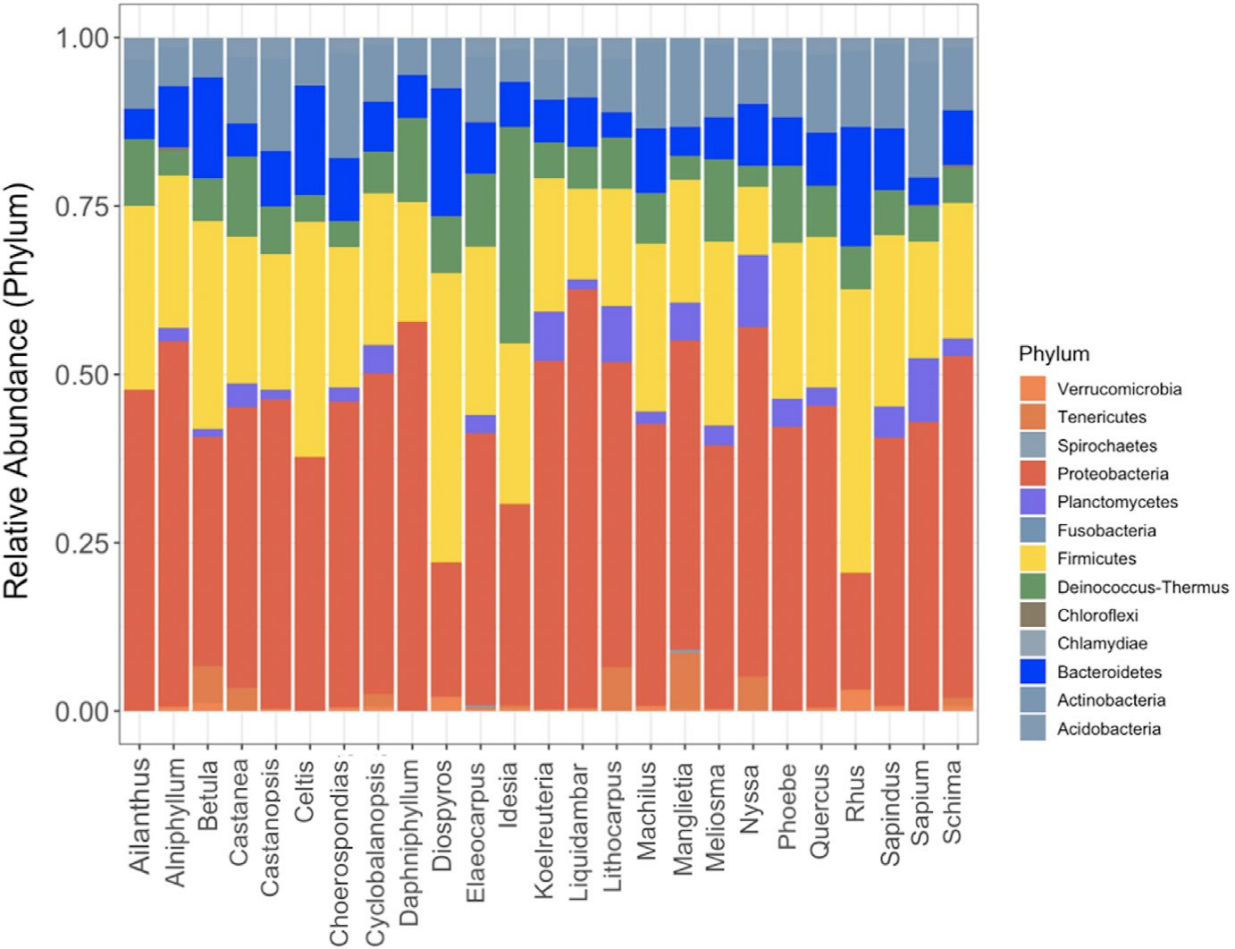
Bacterial phyla present in each tree genus, with relative abundances averaged across tree individuals of the same genus. The bacterial phyla are listed in the legend. Analysis is limited to phyla with site relative abundance >= 0.1%

After merging reads and filtering for quality, we obtained 16,490,248 reads (out of 20,466,051 raw reads) in total from 634 caterpillar individuals, delineated to 7,909 bacterial OTUs. There were minor shifts in bacterial phyla among tree genera of plots (Figure 2 and Table S2). The three most abundant bacterial phyla were Proteobacteria, Firmicutes, and Actinobacteria. Among classes, Alpha‐ and Gamma‐proteobacteria classes predominated. We found six core OTUs (present in more than 90% of the samples), which belong to four phyla (Proteobacteria, Actinobacteria, Firmicutes, Deinococcus‐Thermus). Based on information of the taxonomic assignment, two of them belong to the genus Enterococcus, one was *Enterococcus sp*. and the other was *Enterococcus faecalis,* and the remains were common species exist in the environment. We chose the four most abundant Lepidoptera species (> 24 individuals and observed at all tree diversity levels) to examine distribution patterns of their bacterial phyla (Figure S2). We found that the bacteria of a given Lepidoptera species varied greatly in composition among tree diversity levels, and the bacteria of different Lepidoptera species often varied substantially within a given tree diversity level. Further, based on the assigned taxonomic information, we inferred that only two of the core species are likely to have been derived from the gut of caterpillars, the remainder likely from the leaf surface or elsewhere in the environment.

**FIGURE 3:**
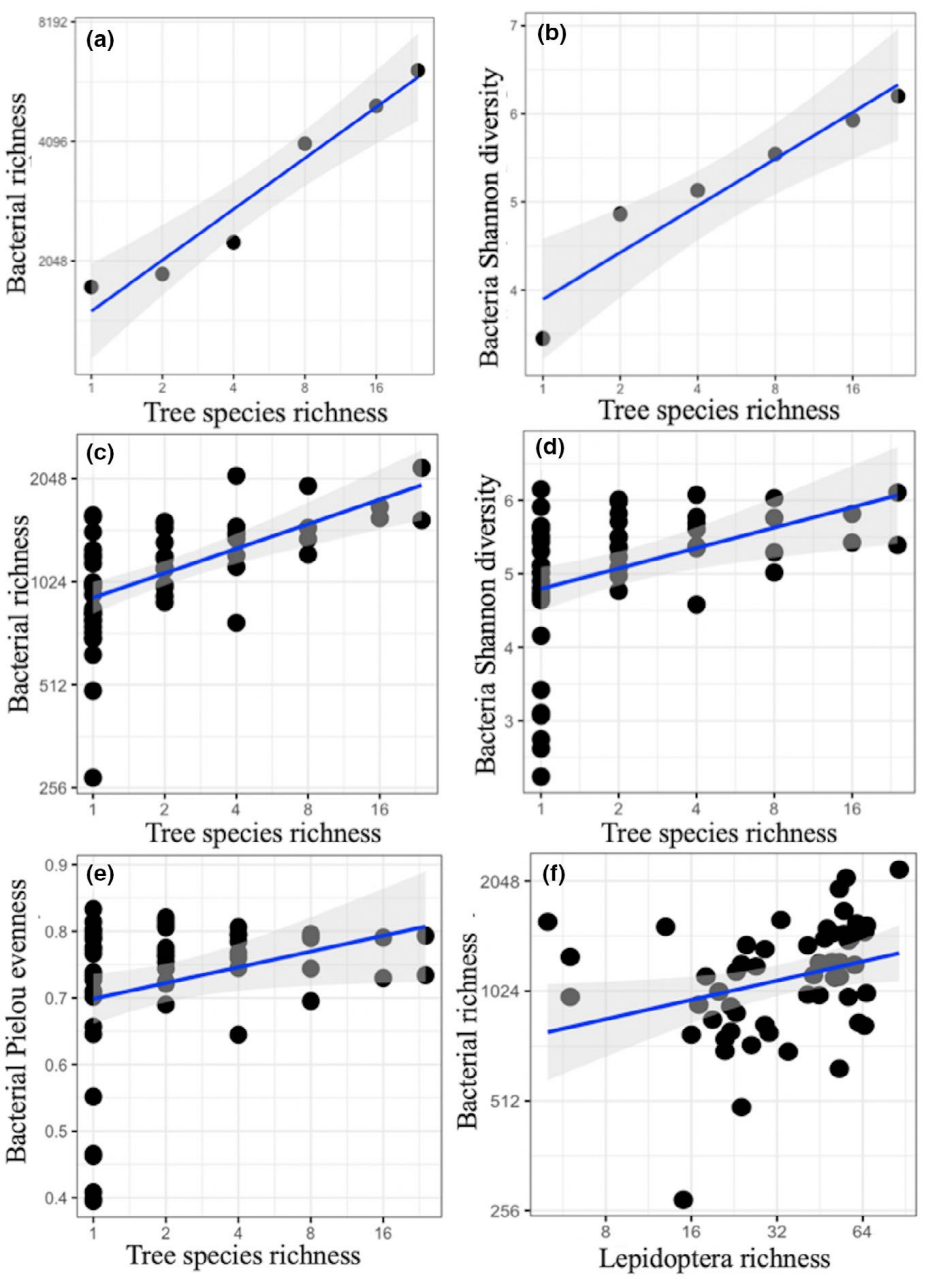
Relationships between (a) tree species richness and bacterial richness, (b) tree species richness and bacterial Shannon diversity (at richness level), (c) tree species richness and observed bacterial richness, (d) tree species richness and bacterial Shannon diversity, (e) tree species richness and bacterial Pielou's evenness, (f) Lepidoptera richness and observed bacterial richness. Regression lines (with 95% confidence bands) show significant (*p *≤ .05) relationships. The axis values are on a log‐scale for tree species richness, Lepidoptera richness and richness of bacteria. Note: Both of observed bacterial richness and estimated bacterial richness were positively correlated with tree species richness, only the former was shown here

Bacterial richness (both S_obs_ and Chao1) and Shannon diversity differed across the study plots, and both were significantly correlated with tree species richness (Figure 3c–3f, Table 2). These results were confirmed when analyzing the relationship between tree species richness and bacterial diversity at the tree richness level (i.e., based on similar numbers of caterpillars in each richness level; Figure 3a and 3b). Furthermore, estimated bacterial richness (Chao1) at the plot level was also affected by Lepidoptera abundance, Lepidoptera richness, and the interaction of tree species richness and Lepidoptera richness (Table 2). Tree species richness also corresponded to the caterpillar microbiomes when using Pielou's evenness. Moreover, bacterial richness (both S_obs_ and Chao1) were positively correlated with CWMs of several leaf traits of tree communities, especially LDMC and leaf K content. Shannon diversity was correlated with tree species richness and the CWMs of LT, leaf Ca content, and K content (Table 2). Pielou's evenness was also positively correlated with CWMs of Ca content and K content.

**TABLE 2:** Summary results of linear models for observed bacterial richness, estimated bacterial richness (Chao1 estimator), Shannon diversity, and Pielou's evenness of bacterial communities across a tree species richness gradient. Standardized parameter estimates (with standard errors, t and p values) are shown for the variables retained in the minimal models

	*Est ± SE*	*t*	*p*
Observed bacterial richness (S_obs_)			
(Intercept)	6.963 ± 0.056	125.067	**<0.001**
Lepidoptera abundance (log)	0.181 ± 0.178	1.017	0.315
Lepidoptera richness (log)	−0.186 ± 0.174	−1.068	0.291
Tree richness (log)	0.205 ± 0.040	5.094	**<0.001**
SiteB	0.149 ± 0.098	1.514	0.137
CWM LDMC	0.140 ± 0.054	2.588	**0.013**
CWM LA	0.087 ± 0.043	2.034	**0.048**
CWM K	−0.134 ± 0.038	−3.484	**0.001**
Lepidoptera abundance: SiteB	−0.341 ± 0.207	−1.649	0.106
Lepidoptera richness: SiteB	0.430 ± 0.200	2.152	**0.037**
Estimated bacterial richness (Chao1)			
(Intercept)	7.340 ± 0.043	170.524	**<0.001**
Lepidoptera abundance (log)	−0.203 ± 0.090	−2.256	**0.028**
Lepidoptera richness (log)	0.276 ± 0.097	2.839	**0.007**
Tree richness (log)	0.220 ± 0.060	3.790	**<0.001**
CWM SLA	−0.075 ± 0.041	−1.856	0.069
CWM K	−0.157 ± 0.039	−4.041	**<0.001**
CWM Mg	0.083 ± 0.039	2.127	**0.038**
Lepidoptera richness: Tree richness	0.101 ± 0.054	1.863	0.069
Shannon diversity			
(Intercept)	1.517 ± 0.036	41.686	**<0.001**
Lepidoptera abundance (log)	−0.136 ± 0.063	−2.179	**0.034**
Lepidoptera richness (log)	0.156 ± 0.067	2.341	**0.023**
Tree richness (log)	0.062 ± 0.027	2.283	**0.027**
SiteB	0.194 ± 0.060	3.239	**0.002**
CWM K	−0.141 ± 0.031	−4.582	**<0.001**
CWM Ca	0.105 ± 0.033	3.209	**0.002**
CWM LT	−0.072 ± 0.027	−2.625	**0.012**
Pielou's evenness			
(Intercept)	−0.412 ± 0.033	−12.348	**<0.001**
Lepidoptera abundance (log)	−0.117 ± 0.056	−2.076	**0.044**
Lepidoptera richness (log)	0.065 ± 0.061	1.068	0.291
Tree richness (log)	0.079 ± 0.032	2.462	**0.018**
SiteB	0.175 ± 0.055	3.153	**0.003**
CWM K	−0.108 ± 0.028	−3.883	**<0.001**
CWM Ca	0.088 ± 0.029	3.071	**0.004**
Lepidoptera richness: SiteB	0.087 ± 0.049	1.752	0.086
Tree richness: SiteB	−0.081 ± 0.049	−1.644	0.107

Abbreviations: CWM Ca, Community‐weighted mean value of leaf Calcium concentration; CWM K, Community‐weighted mean value of leaf potassium concentration; CWM Mg, Community‐weighted mean value of leaf magnesium concentration; CWM LDMC, Community‐weighted mean value of leaf dry matter content; CWM LT, Community‐weighted mean value of leaf toughness; CWM SLA, Community‐weighted mean value of specific leaf area; CWM LA, Community‐weighted mean value of leaf area; FD, Functional diversity.

Bold values mean the corresponding predictors that had a significant effect on certain variable.

**FIGURE 4:**
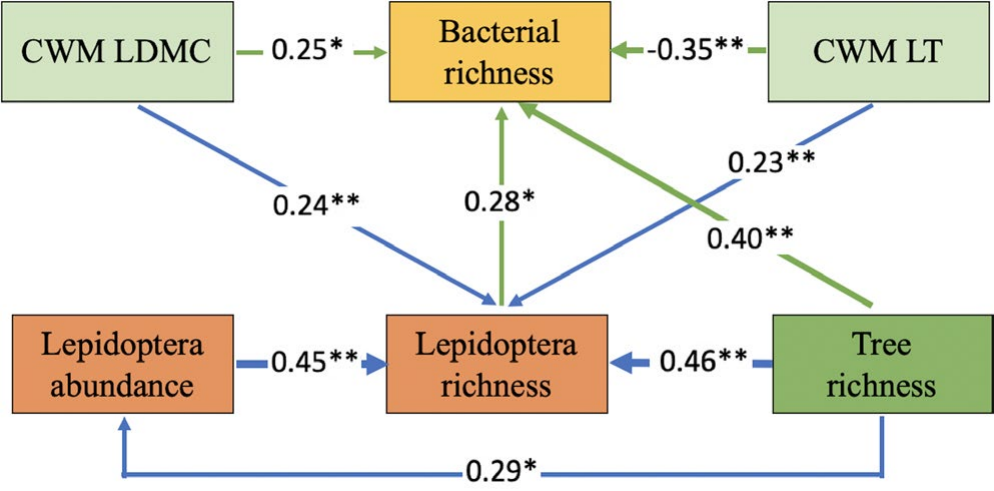
Path model of the effects of tree species richness (direct effect and indirect effects through Lepidoptera abundance and Lepidoptera richness), Lepidoptera richness, CWM LT (direct effect and indirect effect through Lepidoptera richness), CWM LDMC (indirect effect through Lepidoptera richness) on richness of bacterial community. The path coefficients next to the arrows represent the strength of the positive or negative effects of one variable on another (**p < 0.001; *p < 0.05). See Table S1 and S3 for abbreviations and statistical values

The path analyses (Figure 4; Table S3) showed that tree species richness directly influenced Lepidoptera richness, which in turn affected the bacterial richness. At the same time, bacterial richness was driven by tree species richness both independently and directly, and this influence was far greater than that of Lepidoptera richness on bacterial richness. Moreover, leaf traits also negatively affected bacterial richness and had an indirect influence through effects on Lepidoptera richness. The CWM of LDMC had a positive effect on bacterial richness both directly and indirectly, through Lepidoptera richness.

**FIGURE 5:**
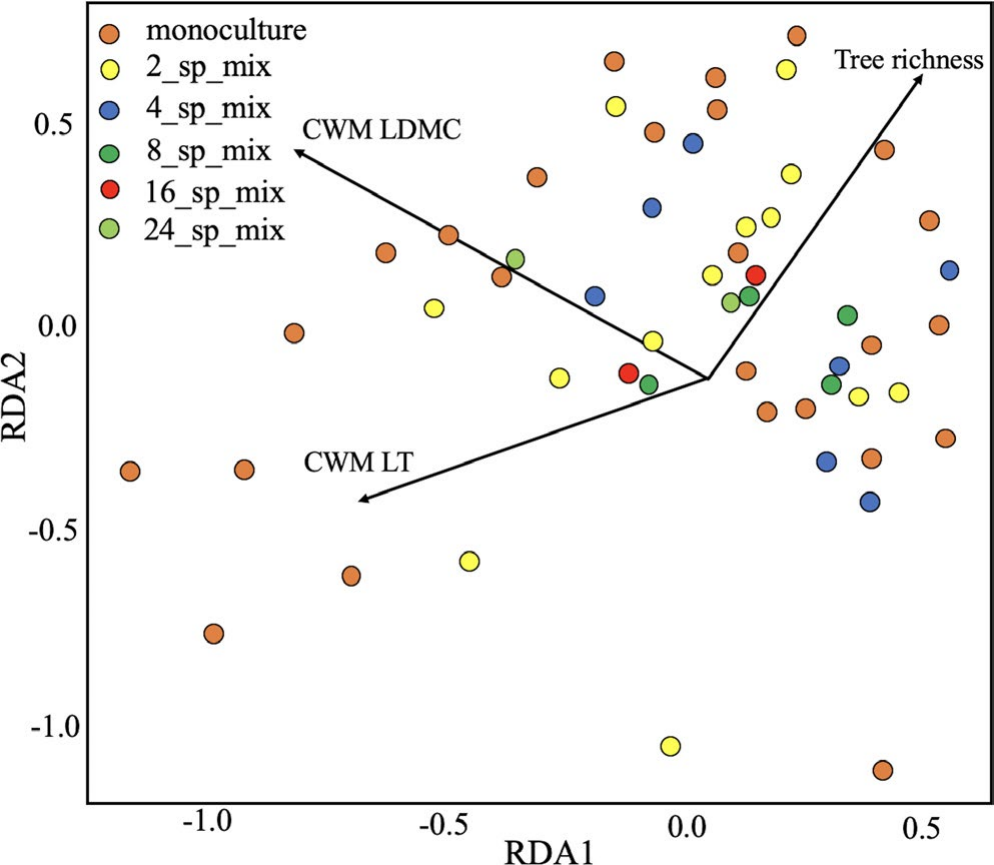
Distance‐based redundancy analysis plot showing the relationships of CWM LDMC, CWM LT, and tree richness to the bacterial community structure. The plot represents db‐RDA analysis based on Bray–Curtis distance with all of the plot covariables and CWM of leaf traits as explanatory variables. CWM LDMC, CWM LT, and tree richness were three significant explanatory variables (*p* < .05)

The bacterial Beta diversity among different tree diversity gradients was significantly different (Jaccard dissimilarity, *F* = 1.946, *p* =.001; Morisita dissimilarity: *F* = 2.245, *p* =.04; Horn–Morisita dissimilarity: *F* = 2.237, *p* =.03; Bray–Curtis dissimilarity: *F* = 2.004, *p* =.004; Chao dissimilarity: *F* = 0.316, *p* =.007). Mantel test indicated that there was no significant relationship between bacterial composition and spatial location (r = 0.07, *p* =.14). db‐RDA analysis was performed to determine whether plot covariables and CWM of leaf traits affected the bacterial community structure of lepidopteran samples. The bacterial community structure differed across the study plots but was not significantly affected by species richness of Lepidoptera. However, it was influenced by tree richness (db‐RDA pseudo‐*F* = 1.41, Padj < 0.05; Figure 5), CWM LDMC (db‐RDA pseudo‐*F* = 2.51, Padj < 0.01), and CWM LT (db‐RDA pseudo‐*F* = 1.88, Padj < 0.01).

**FIGURE 6:**
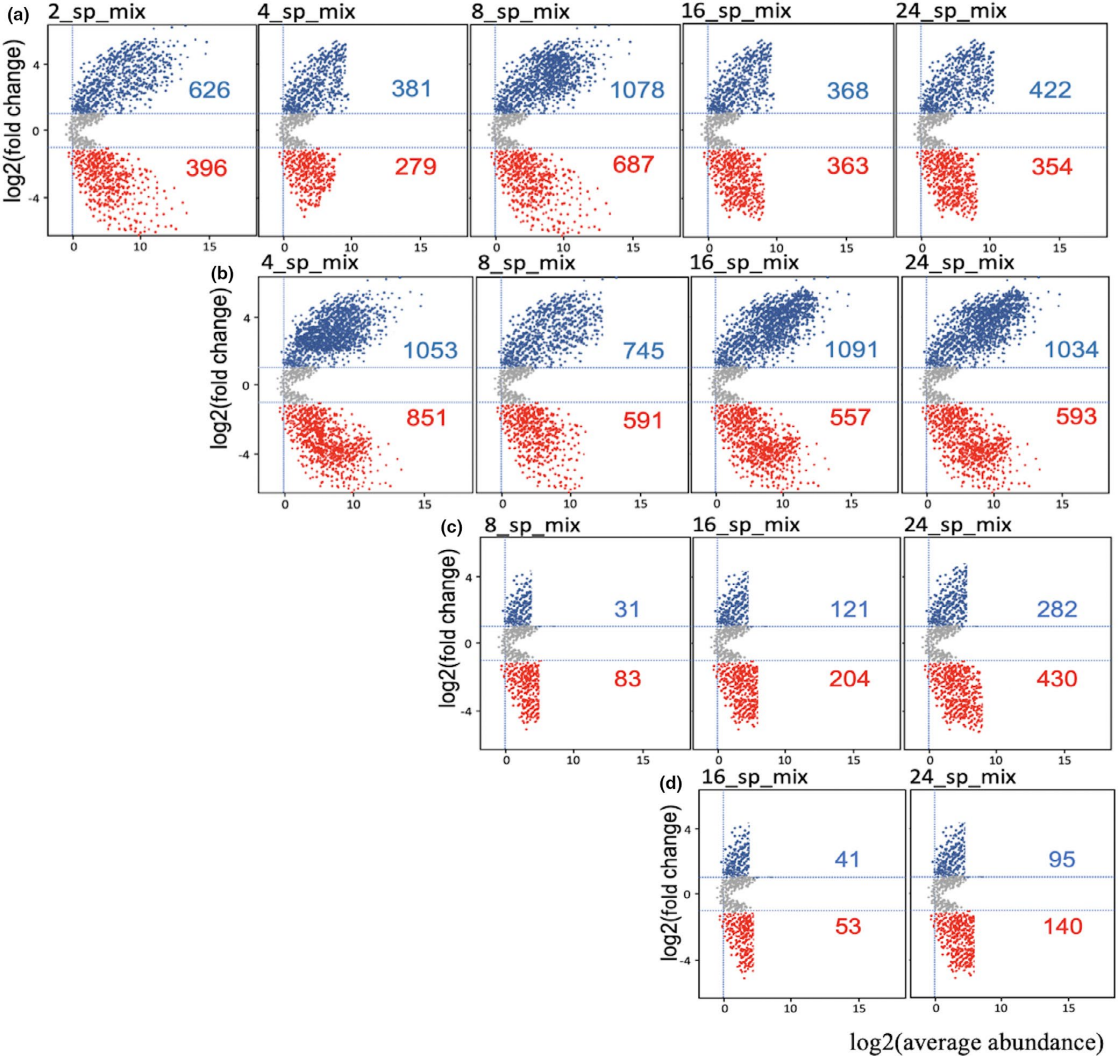
Differentially enriched bacterial OTUs across tree species richness levels. We firstly used bacterial counts from monocultural plots as a control and compared it with the bacterial counts from 2, 4, 8, 16, and 24 species mixed plots successively. Part (a) to (d) represent the results of using the monocultures to 8 species mixtures as a control separately. Each point represents an individual species, and the position along the y‐axis represents the abundance fold change compared with the control

The analyses of differentially abundant bacterial OTUs between tree richness levels were conducted by fitting a generalized linear model with a negative binomial distribution to normalized values for each of the 7,909 bacterial OTUs, and testing for differential abundance using a likelihood ratio test. We first used bacterial counts from monoculture plots as a control and an adjusted P value cutoff of 0.01, and compared it with the bacterial counts from 2, 4, 8, 16, and 24 species mixed plots separately. As shown in Figure 6, the enriched bacterial counts were always greater than depleted counts in comparison with the control. Then, we used the counts from 2, 4, 8, and 16 tree species mixtures as a control and compared successively with higher diversity mixtures. We found that the counts of the enriched species were higher than that of depleted species with increasing diversity when using monocultures and 2 species mixtures as controls. Although when using the 4‐species mixtures as a control, the counts of depleted bacteria exceeded the enriched, and the counts of both reduced significantly. In addition, Figure S3 shows the numbers of differentially enriched and depleted bacterial OTUs between each tree richness level compared with different controls.

## 4 DISCUSSION

This study highlights the impacts of tree diversity on the diversity and community composition of herbivore‐associated bacteria, and shows they are influenced by tree diversity and characteristics of the leaf, in what is both a direct and indirect interaction. The direct effect of tree diversity on bacterial diversity was found to predominate, whereas the composition of bacterial communities was to a large part determined by tree diversity and leaf functional traits, especially LDMC and LT, but also chemical leaf traits such as calcium and potassium concentrations. Considering that there is an increasing number of studies reporting that the larva of Lepidoptera recruit microbes from the environment, and that they lack a persistent gut microbiome (Hammer et al., 2017; Hammer et al., 2019), these are key findings that help to better understand which environmental factors determine these microbial communities and how such environmental effects may influence herbivore functioning.

Tree diversity affected the diversity of caterpillar‐associated bacteria through influencing the abundance and diversity of lepidopteran larvae. Wang et al. (2019) reported that the impact of tree diversity on herbivore diversity is generally indirect, as tree diversity had strong effects on herbivore abundances, which in turn can affect herbivore diversity. The increase in bacterial diversity that follows increasing Lepidoptera diversity at the plot level is consistent with the expectation that more Lepidoptera individuals and species provide more niche opportunities for bacteria (Akiko et al., 2015). Thus, diversity at one trophic level begets biodiversity at other trophic levels. Moreover, tree diversity was found to also directly influence the diversity of caterpillar‐associated bacteria. The most common bacterial groups of the phyllosphere are Acidobacteria, Actinomycetes, Bacteroidetes, Firmicutes, and Proteobacteria (Bodenhausen et al., 2013; Bulgarelli et al., 2013), the latter both the most abundant taxonomic group observed in our study as well as generally associated with the phyllosphere reported by others (Humphrey et al., 2014). This suggests that the phyllosphere is one of the main sources of the herbivore microbiome (Hammer et al., 2017; Whitaker et al., 2016). Kembel et al. (2014) reported that phyllosphere microbial composition differs according to position and height of tree leaves, and physiological and biochemical features such as water content, leaf mass, nitrogen and phosphorus concentrations, leaf surface structure, and thickness. Moreover, both bacterial and fungal communities of the phyllosphere are seasonally dynamic (Jumpponen & Jones, 2010; Rastogi et al., 2012). We suspect that diversity in environmental drivers is one of the main reasons for the very high bacterial compositional dissimilarity observed between caterpillars in this study site, with merely 6 bacteria OTU of 7,909 that were commonly observed. Further, taxonomic analysis showed that only two of these core species originate from the caterpillar gut, the remainder presumably from the leaf surface or elsewhere in the environment. This result is generally consistent with the finding reported by Hammer et al. (2017), Hammer et al. (2019), that resident microbial symbionts are generally absent, or present in low numbers, in the caterpillar gut. Our result implies a correspondence between herbivore‐associated microbes and their host plants. Another question which remains to be tested is the long‐term stability of phyllosphere to herbivore microbial interaction, and to what degree they are altered upon herbivores encountering new host plants through movement or feeding.

It is important to note the effect of leaf traits on the diversity and distribution of caterpillar‐associated bacteria. As mentioned above, the phyllosphere appears to be a key source for herbivore microbiomes and is moderated by tree characteristics such as leaf structure (also, LDMC is directly affected by leaf thickness, structure, and specific leaf area and reflects the ability of plants to obtain resources). LDMC and LT are usually expected to negatively associate with herbivory because structurally robust leaves are relatively difficult to consume (Pérez et al., 2003). However, both the results herein and some previous reports suggest a positive relationship between LDMC and leaf herbivory (Lepidoptera richness in this study; Schuldt et al., 2012), probably because there are herbivores specifically adapted to tough leaves (Pérez et al., 2003) and herbivores have to consume more of less nutritious foliage to gain the same nitrogen accumulation rates (Scriber & Slansky, 1981).

In addition to the two leaf traits (LDMC and LT) mentioned above, we also found that leaf potassium (K) content, calcium (Ca) content, and magnesium (Mg) content can affect bacterial richness, Shannon diversity, or Pielou's evenness of the bacteria community; thus, there are potential links between leaf traits, herbivores, and their associated microbes. Compared to other leaf traits, potassium (K) content, calcium (Ca) content, and magnesium (Mg) content have received little attention with respect to herbivory, but some studies have shown that they can have either positive or negative impacts on herbivore insects (e.g., fecundity; Awmack & Leather, 2002). There is, however, considerable variation in mineral requirements of herbivore insects.

Tree species richness was found to be an important factor that affected caterpillar‐associated bacteria community composition. A remarkable result was that certain bacterial OTUs were more abundant in tree species mixtures compared to monoculture plots. However, the accumulation rate of bacterial taxa in more species‐rich mixtures gradually decreased. That the increase of tree diversity might have a certain stabilizing effect on the herbivore‐associated bacterial community was also supported by our finding that the bacterial species composition became more homogenous with tree species richness. From this, we would conclude that more tree species‐rich forests might have richer but more stable and homogeneous bacterial communities.

We conclude that tree diversity and leaf traits of the tree community are strong drivers of the caterpillar‐associated bacteria communities in our subtropical forest. Our study revealed the linkages between tree (leaves), herbivore insects, and herbivore‐associated microbes, which contributes to develop a more comprehensive understanding of relationship between herbivores and their environment. Moreover, the driving and stabilizing effects of tree diversity on herbivore‐associated bacteria suggest that future research should take effects of plants on herbivore‐associated microbes into consideration, when studying the relationships between plants and herbivores.

## CONFLICT OF INTEREST

The authors declare no conflict of interest.

## AUTHOR CONTRIBUTION

**Yi Li:** Methodology (lead); Writing‐original draft (lead). **Douglas Chesters:** Methodology (supporting); Writing‐review & editing (supporting). **Ming‐Qiang Wang:** Methodology (equal). **Tesfaye Wubet:** Formal analysis (supporting); Writing‐original draft (supporting). **Andreas Schuldt:** Formal analysis (supporting); Writing‐original draft (supporting). **Perttu Anttonen:** Methodology (supporting); Writing‐review & editing (supporting). **Peng‐Fei Guo:** Methodology (supporting); Writing‐original draft (supporting). **Jing‐Ting Chen:** Methodology (equal); Writing‐original draft (equal). **Qingsong Zhou:** Methodology (supporting); Writing‐original draft (supporting). **Nai‐Li Zhang:** Formal analysis (supporting); Writing‐original draft (supporting). **Keping Ma:** Methodology (supporting); Writing‐original draft (supporting). **Helge Bruelheide:** Formal analysis (supporting); Writing‐original draft (supporting). **Chun‐Sheng Wu:** Methodology (supporting); Writing‐original draft (supporting); Writing‐review & editing (supporting). **Chao‐Dong ZHU:** Project administration (lead); Supervision (lead).

## AUTHOR CONTRIBUTIONS

CDZ: Idea conception for the manuscript. YL: Research design. YL, DC, MQW, TW, AS, PFG, JTC, PA, NLZ, QSZ, CSW, and KPM: Data collection and/or data contribution and advice. YL: Bioinformatic analyses and the statistical analyses and wrote the manuscript, with input by AS and DC, with input from all coauthors.

## Supporting information

Supplementary Material

## Data Availability

Caterpillar‐associated bacterial sequence data can be downloaded from NCBI, accession Number: PRJNA698461. The data of the plot information that support the findings of this study are available from the corresponding author upon reasonable request.
