# Supplementary material for: Tree diversity and functional leaf traits drive herbivore‐associated microbiomes in subtropical China
Source: Ecol Evol. 2021 Mar 31;11(11):6153–66. doi: 10.1002/ece3.7434 (PMC8207151; doi:10.1002/ece3.7434)
Supplement: Supplementary file 1 — Supplementary Material [file ECE3-11-6153-s001.docx]

Table S1. The list of leaf traits and corresponding abbreviations.

| Morphological leaf traits | |
| --- | --- |
| Leaf area | LA |
| Specific leaf area | SLA |
| Leaf dry matter content | LDMC |
| Leaf toughness | LT |
| Chemical leaf traits | |
| Leaf potassium content | K |
| Calcium content | Ca |
| Magnesium content | Mg |
| Sodium content | Na |
| Phosphorus content | P |
| Carbon content | C |
| Nitrogen content | N |
| C: N ratio of leaf | C: N |

Table S2. Relative abundance of bacterial phyla as correlated with tree genus. Cells are shaded (yellow to dark red) relative to bacterial phylum abundance. The tree genera name in upper part belong to the same family, and the same color covered on tree genera name in lower part represent genera from the same family.


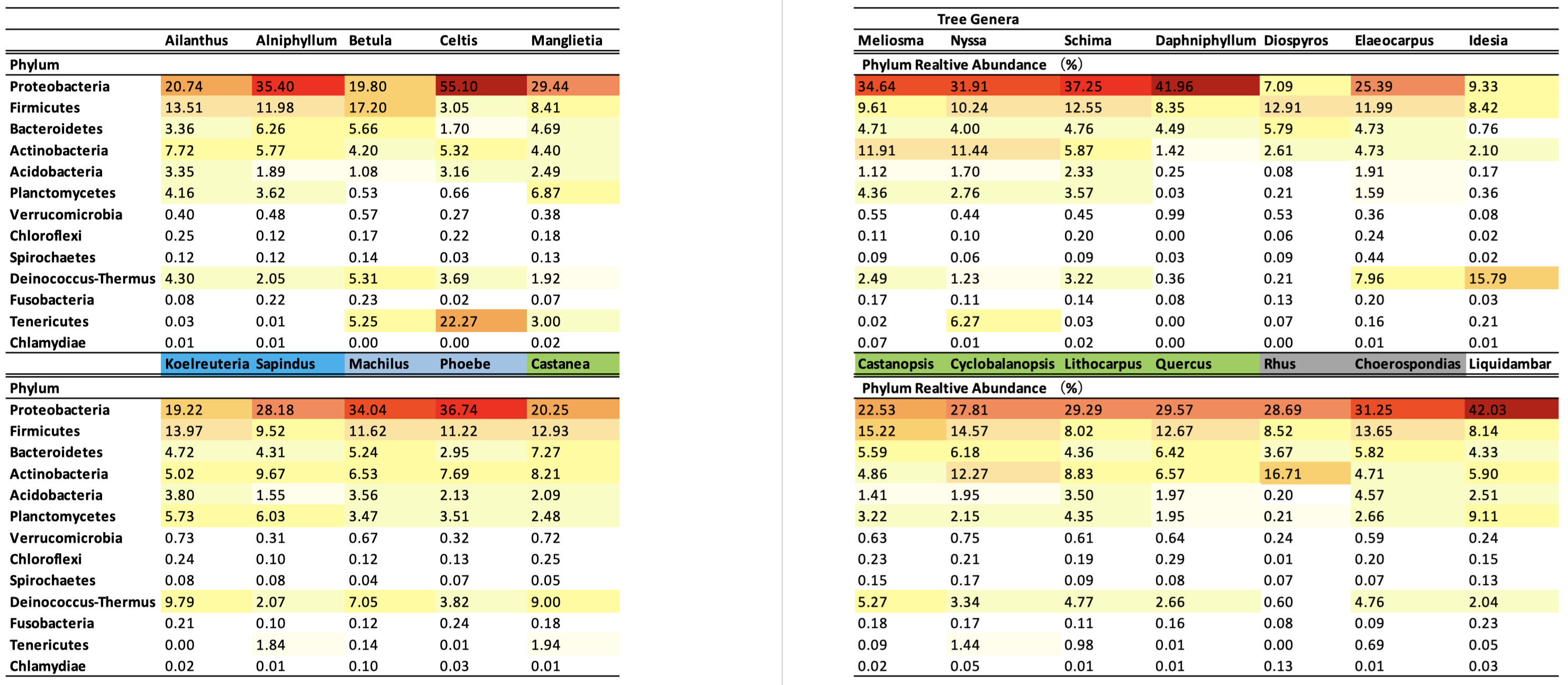

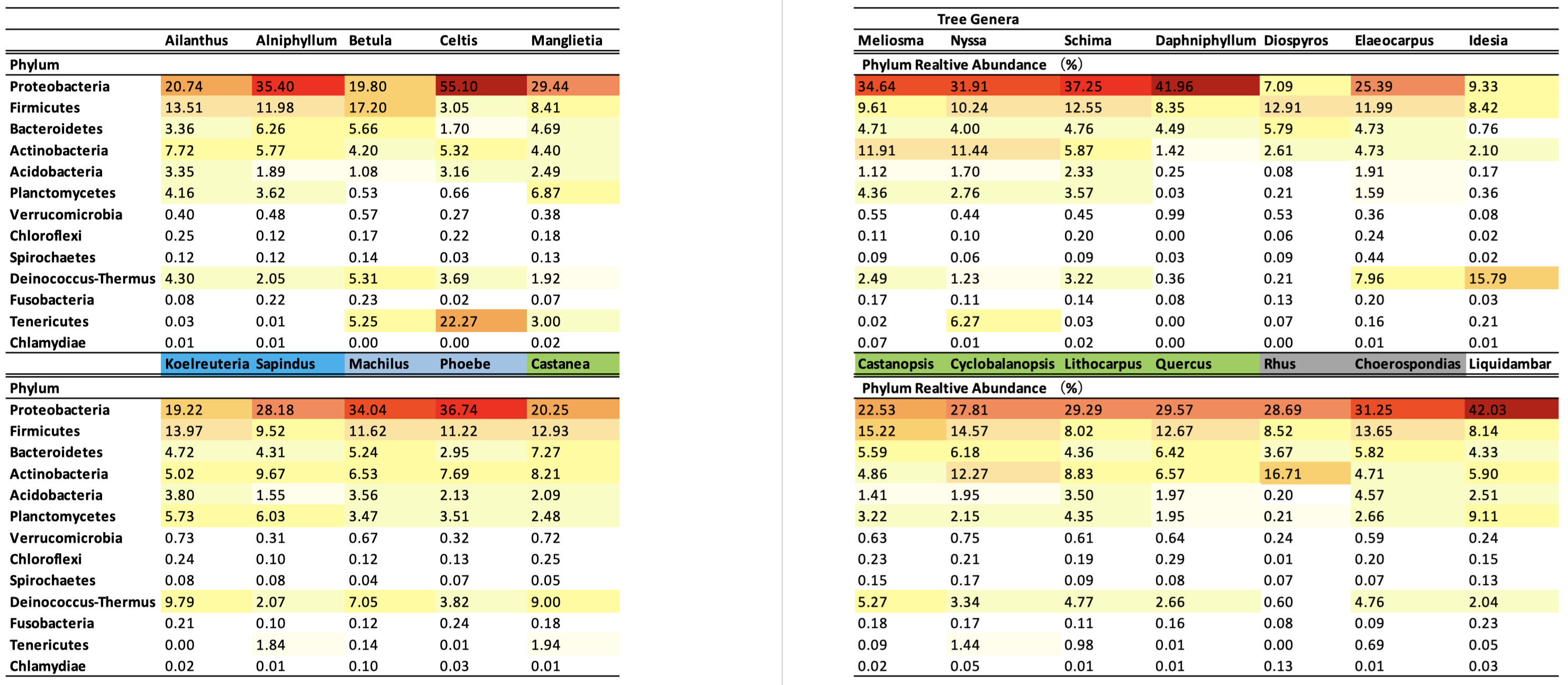


Table S3. Path model results of the effects of tree species richness (direct effect and indirect effect through Lepidoptera abundance and Lepidoptera richness), Lepidoptera richness, CWM LT (direct effect and indirect effect through Lepidoptera richness), CWM LDMC (indirect effect through Lepidoptera richness) on richness of bacteria community.

| **Model** |  | | |  |  |
| --- | --- | --- | --- | --- | --- |
| Estimator | Maximum likelihood | | |  |  |
| Comparative Fit Index (CFI) | 0.978 | | |  |  |
| Tucker-Lewis Index (TLI) | 0.914 | | |  |  |
| Number of observations | 54 | | |  |  |
| χ 2 | 6.039 | | |  |  |
| RMSEA | 0.137 | | |  |  |
| Degrees of freedom | 3 | | |  |  |
| P (Chi-square) | 0.111 | | |  |  |
|  |  |  |  |  |  |
| **Regressions** |  |  |  |  |  |
| Response ~ Predictor | Estimate | SE | z | P | Std. Estimate |
| **Lepidoptera richness ~** |  |  |  |  |  |
| Tree richness | 1.518 | 0.285 | 5.321 | < .001 | 0.464 |
| **Lepidoptera abundance ~** |  |  |  |  |  |
| Tree richness | 5.630 | 2.489 | 2.262 | 0.024 | 0.294 |
| **Lepidoptera richness ~** |  |  |  |  |  |
| Lepidoptera abundance | 0.077 | 0.015 | 5.205 | < .001 | 0.450 |
| CWM LT | 21.645 | 8.275 | 2.616 | 0.009 | 0.230 |
| CWM LDMC | 0.069 | 0.025 | 2.701 | 0.007 | 0.237 |
| **Bacterial richness ~** |  |  |  |  |  |
| CWM LDMC | 1.690 | 0.715 | 2.362 | 0.018 | 0.253 |
| CWM LT | -761.709 | 231.647 | -3.288 | < .001 | -0.352 |
| Lepidoptera richness | 6.431 | 2.985 | 2.154 | 0.031 | 0.280 |
| Tree richness | 30.087 | 9.269 | 3.211 | < .001 | 0.400 |
|  |  |  |  |  |  |
| **Variances** |  |  |  |  |  |
| Variable | Estimate | SE | z | P | Std. Estimate |
| Lepidoptera richness | 104.207 | 20.055 | 5.196 | < .001 | 0.369 |
| Lepidoptera abundance | 8805.170 | 1694.556 | 5.196 | < .001 | 0.913 |
| Bacterial richness | 75313.043 | 14494.002 | 5.196 | < .001 | 0.506 |
|  |  |  |  |  |  |
| **R-Square** |  |  |  |  |  |
| Variable | Estimate |  |  |  |  |
| Lepidoptera richness | 0.631 |  |  |  |  |
| Lepidoptera abundance | 0.087 |  |  |  |  |
| Bacterial richness | 0.494 |  |  |  |  |

Note: CWM LDMC, Community-weighted mean value of leaf dry matter content; CWM LT, Community-weighted mean value of leaf toughness.


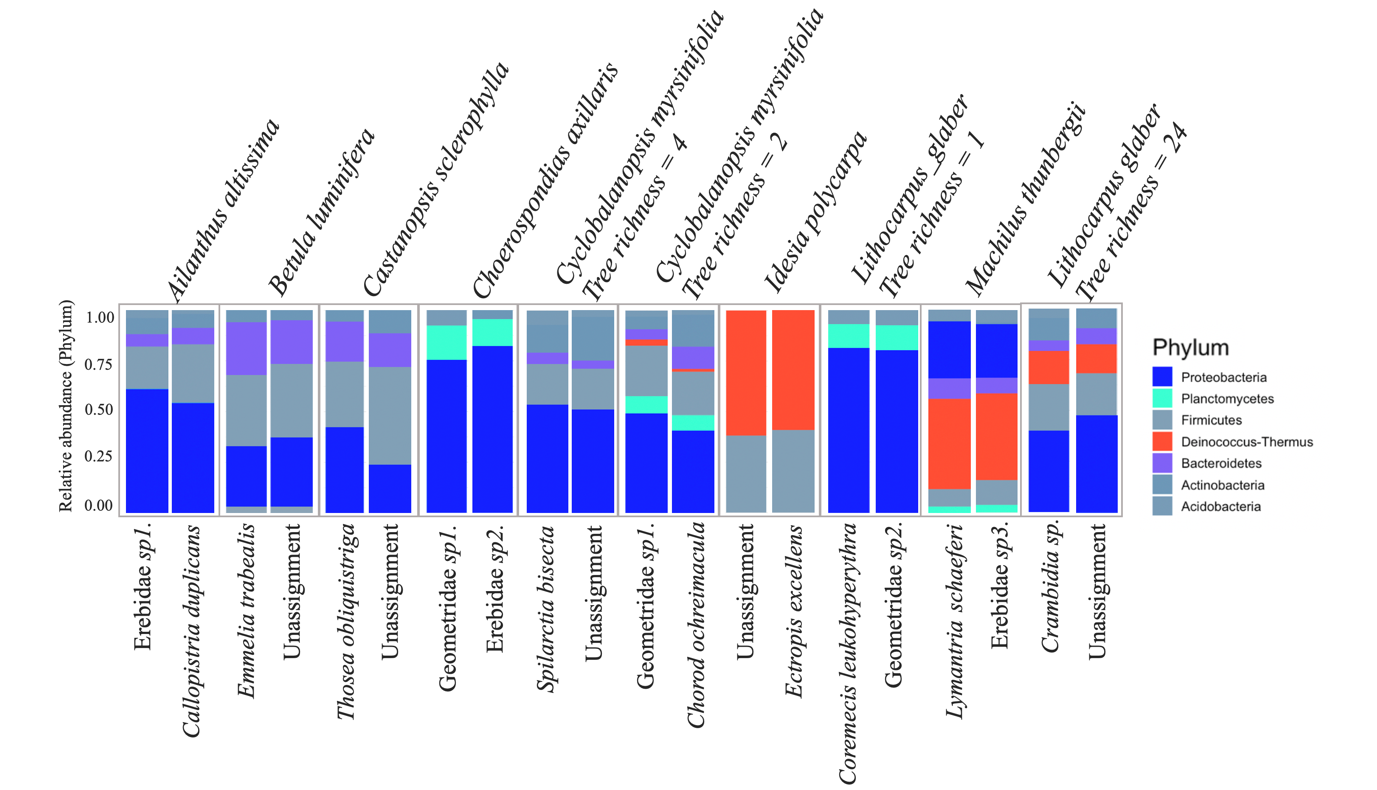
Figure S1. Relative abundance of individual phyla that correlated with Lepidoptera species in this study (the x axis represents the Lepidoptera species, and that species name in the same frame indicate that they come from the same tree species in the same plot). Analysis is limited to phyla with relative abundance >= 0.1%.

**
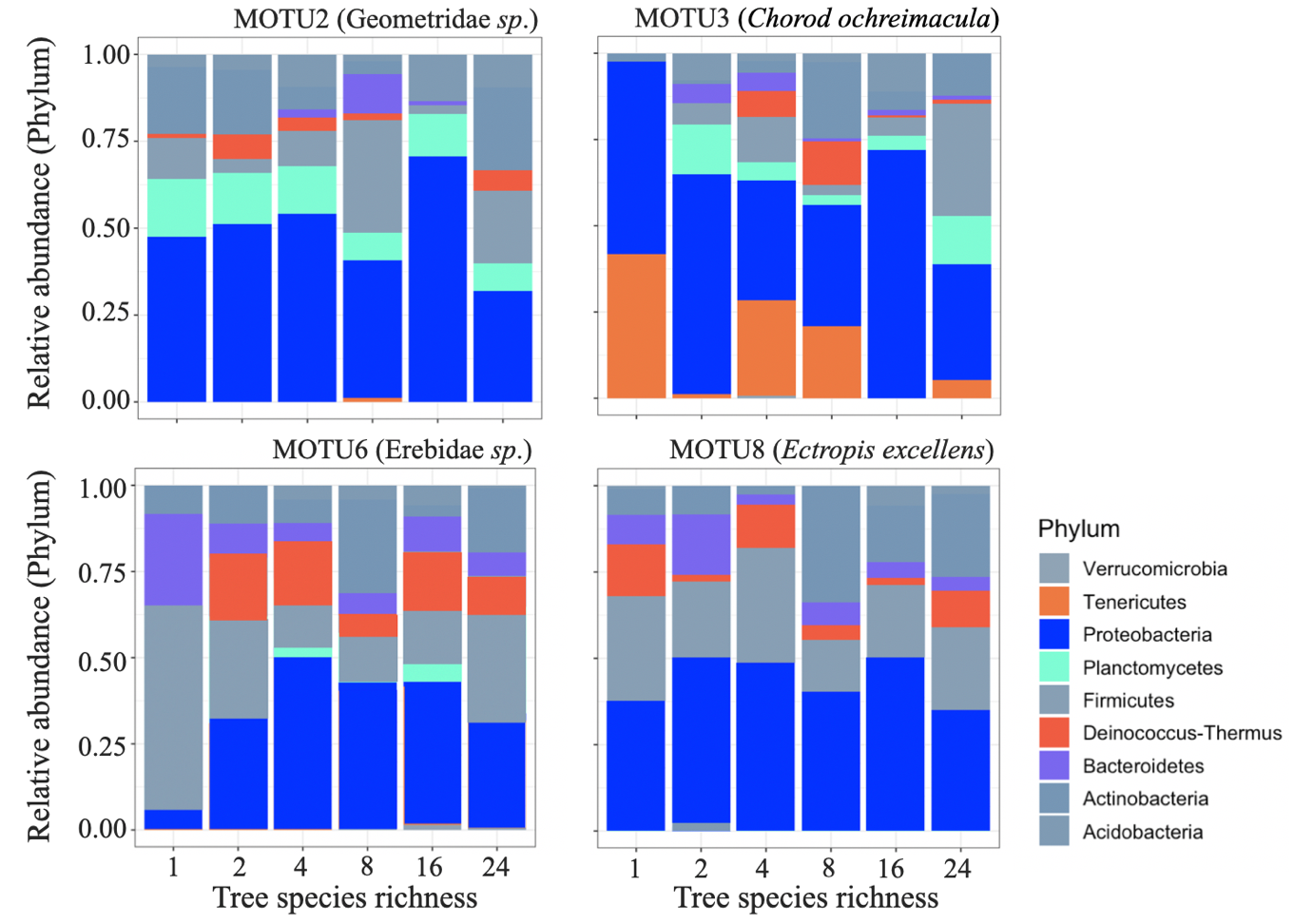
**Figure S2. Relative abundance of individual phyla that correlated with 4 most abundant Lepidoptera species in this study (individual number >= 24 and distributed in all diversity levels). Analysis is limited to phyla with relative abundance >= 0.1%.


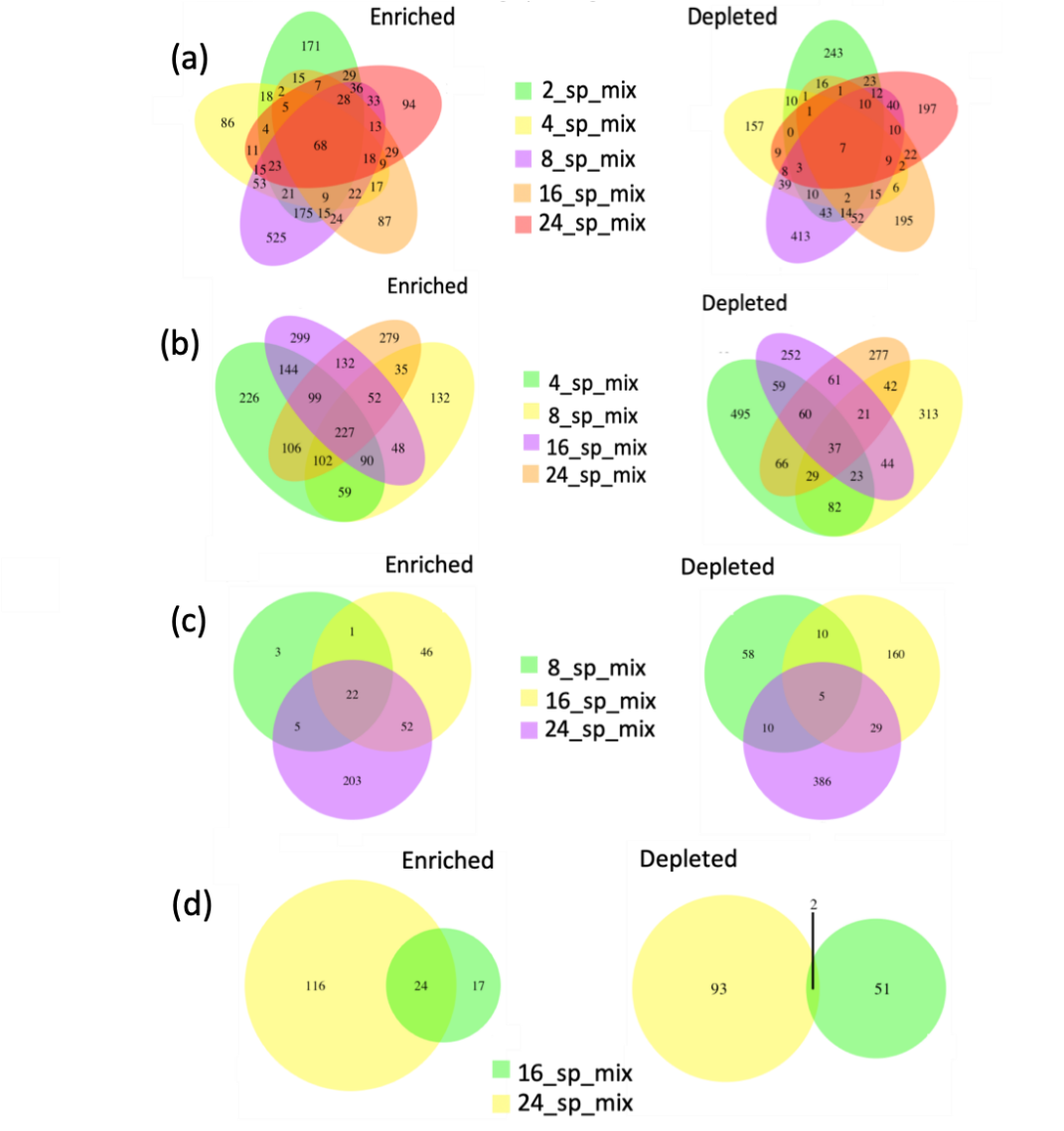
Figure S3. Numbers of differentially enriched and depleted bacterial OTUs between each tree richness level compared with different controls. Part (a) to (d) represent the results of using the monocultures to 8 species mixtures as a contr
